# Myocardial lipid content in Fabry disease: a combined ^1^H-MR spectroscopy and MR imaging study at 3 Tesla

**DOI:** 10.1186/s12872-016-0382-4

**Published:** 2016-10-28

**Authors:** B. Petritsch, H. Köstler, A. M. Weng, M. Horn, T. Gassenmaier, A. S. Kunz, F. Weidemann, C. Wanner, T. A. Bley, M. Beer

**Affiliations:** 1 Department of Diagnostic and Interventional Radiology, University Hospital Würzburg, Oberdürrbacher Straße 6, 97080 Würzburg, Germany; 2 University of Würzburg, Comprehensive Heart Failure Center, 97080, Würzburg, Germany; 3 Department of Internal Medicine II/Cardiology, Katharinen-Hospital Unna, Obere Husemannstr.2, 59423 Unna, Germany; 4 Department of Internal Medicine I, University Hospital Würzburg, Oberdürrbacher Straße 6, 97080 Würzburg, Germany; 5 Department of Diagnostic and Interventional Radiology, University Hospital Ulm, Albert-Einstein-Allee 23, 89081, Ulm, Germany

**Keywords:** Morbus Fabry, Magnetic resonance spectroscopy, Myocardial lipid content, Rare diseases, Lysosomal storage disease, Late gadolinium enhancement

## Abstract

**Background:**

Fabry disease is characterized by a progressive deposition of sphingolipids in different organ systems, whereby cardiac involvement leads to death. We hypothesize that lysosomal storage of sphingolipids in the heart as occurring in Fabry disease does not reflect in higher cardiac lipid concentrations detectable by ^1^H magnetic resonance spectroscopy (MRS) at 3 Tesla.

**Methods:**

Myocardial lipid content was quantified in vivo by ^1^H-MRS in 30 patients (12 male, 18 female; 18 patients treated with enzyme replacement therapy) with genetically proven Fabry disease and in 30 healthy controls. The study protocol combined ^1^H-MRS with cardiac cine imaging and LGE MRI in a single examination.

**Results:**

Myocardial lipid content was not significantly elevated in Fabry disease (*p* = 0.225). Left ventricular (LV) mass was significantly higher in patients suffering from Fabry disease compared to controls (*p* = 0.019). Comparison of patients without signs of myocardial fibrosis in MRI (LGE negative; *n* = 12) to patients with signs of fibrosis (LGE positive; *n* = 18) revealed similar myocardial lipid content in both groups (*p* > 0.05), while the latter showed a trend towards elevated LV mass (*p* = 0.076).

**Conclusions:**

This study demonstrates the potential of lipid metabolic investigation embedded in a comprehensive examination of cardiac morphology and function in Fabry disease. There was no evidence that lysosomal storage of sphingolipids influences cardiac lipid content as measured by ^1^H-MRS. Finally, the authors share the opinion that a comprehensive cardiac examination including three subsections (LGE; ^1^H-MRS; T_1_ mapping), could hold the highest potential for the final assessment of early and late myocardial changes in Fabry disease.

## Background

Fabry-Anderson disease is a x-linked lysosomal storage disease caused by a deficiency of the enzyme *alpha*-galactosidase A (α-Gal A; also termed *ceramide trihexosidase*) [1]. As a consequence, the decreased or almost absent enzymatic activity leads to progressive accumulation and deposition of sphingolipids, mainly Globotriaosylceramide (also termed *ceramide trihexoside*), in different organ systems [2]. The disease is pan ethnic and incidence estimates ranges from about 1 in 40.000 up to 60.000 [1, 3]. Although the disease predominantly affects males, it is now widely accepted that females - as carriers of the disease - can also be affected to a mild or even severe degree due to random X-chromosomal inactivation [4, 5].

Cardiac involvement is crucial for morbidity and mortality of the disease. Hallmarks of cardiac involvement in Fabry disease are myocardial hypertrophy and fibrosis. Both pathologies have been previously studied by magnetic resonance imaging, using cine- and late gadolinium enhancement (LGE)- sequences [6–8].

Several theories for the genesis of cardiac alterations in Fabry disease are being discussed [9, 10]. It is widely accepted that fibrosis is a key feature of Fabry disease. Currently, late and secondary myocardial changes (e.g. LGE or perfusion mismatch) are used to plan enzyme replacement therapy (ERT) as the current cornerstone of the therapeutic strategy in Fabry disease [9, 11, 12]. Additionally, recent studies revealed the high potential of noncontrast T_1_ mapping as a sensitive and specific cardiovascular MRI parameter in patients with Fabry disease irrespective of sex, left ventricular (LV) morphology and function [13, 14]. Another contributing factor might be cardiac lipid storage, which can be found at different locations in the heart: On the one hand there is intramyocardial triglyceride (TG) deposition, which is linked to various pathological conditions (e.g. diabetes mellitus, metabolic syndrome and obesity); on the other hand there is a lysosomal storage of sphingolipids, especially occurring in Fabry disease. Post-mortem studies, as well as invasive myocardial biopsies have shown a ubiquitous deposition of sphingolipids in the myocardium of patients suffering from cardiac Fabry disease [15].

Due to the fact that ERT is less efficacious when initiated after the development of tissue injury [16], it seems to be of utmost importance to identify potential early predictors of myocardial involvement in Fabry disease, and thus allowing more accurate, respectively early onset of ERT. Therefore, the direct measurement of cardiac lipid deposition has been discussed as a future concept [17]. The highly invasive tissue acquisition by myocardial biopsy is naturally associated with a non-negligible risk of complications in each individual patient [18, 19]. In contrast, non-invasive assessment of myocardial lipid depositions by localized ^1^H magnetic resonance spectroscopy (^1^H-MRS) is a suitable and time-efficient alternative [20]. In the last decade, several studies demonstrated the potential of ^1^H-MRS at 1.5 Tesla (T) for the non-invasive assessment of myocardial lipid content [20–22]. However, results are still limited by the low signal/noise-ratio (SNR) of metabolite resonances at 1.5 T. As the signal strength of all metabolites is proportional to field strength, higher magnetic field strength enable measurements of metabolite concentrations with higher accuracy and without substantial changes of the noise level [23, 24]. The increased availability of 3 T MR systems for clinical routine has opened the field for even more precise non-invasive evaluation of myocardial lipids. In the light of technical development, dual triggered (ECG and respiration) ^1^H-MRS enables the use of this technique for cardiac examinations [25].

We hypothesize that lysosomal storage of sphingolipids as occurring in Fabry disease does not reflect in higher cardiac lipid concentrations detectable by ^1^H-MRS. Therefore the aim of this study was to non-invasively evaluate myocardial lipid deposition in Fabry disease with ^1^H-MRS at 3 T. We designed a one stop cardiac examination to evaluate myocardial lipid content in vivo, left ventricular mass and function, as well as presence or absence of myocardial fibrosis. The study protocol combined ^1^H-MR single voxel spectroscopy with cardiac cine and late enhancement MRI in a single examination.

## Methods

A total of thirty consecutive patients (12 male, 18 female; mean age ± standard deviation (SD), 41.5 ± 13.5 years; range 17–68 years) with genetically proven Fabry disease were included in the study over 24 months. Any history of cardio-vascular disease, diabetes mellitus and respectively any manifestations of an advanced metabolic syndrome were exclusion criteria. 18 patients were treated with ERT at the time of examination (average time of ERT 6.2 years). Body mass index (BMI) ranged between 17.5 and 38.7 kg/m^2^ (mean 24.5 ± 5.5 kg/m^2^).

Thirty healthy volunteers (22 male, 8 female; mean age 30.6 ± 7.7 years; range 24–48 years) without any history of cardio-vascular or metabolic disease were recruited for the control group. BMI varied between 16.0 and 33.5 kg/m^2^ (mean 22.4 ± 3.4 kg/m^2^) in the control group. All participants had to comply with a fasting period of at least 2 h before ^1^H-MRS.

All patients and volunteers were informed about the research status of the study and signed their informed consent. The study protocol was approved by the institutional review board.

All cardiac imaging including ^1^H-MRS was acquired in supine position. Double-triggered high-field cardiac ^1^H MR-spectroscopy and MR-imaging were performed using a 12–channel phased array coil on a 3 T MR scanner (MAGNETOM® Trio, Siemens AG Healthcare Sector, Erlangen, Germany). The time gap between intravenous contrast administration and LGE acquisition was used to acquire the spectroscopic data in the patients cohort. In the control group ^1^H-MRS and cardiac function data were also acquired but no intravenous contrast medium was applied.

### Cardiac MRI

Cine MRI of the short and long heart axis was performed using a Steady-State Free Precession (SSFP) sequence (field of view [FOV] adjusted individually; matrix size, 256 × 216; TE, 1.48 ms; TR, 3.5 ms; flip angle, 45°; slice thickness, 8 mm; slice gap, 0.00 mm; temporal resolution 46.9 ms). The LV mass and LV function, expressed as ejection fraction (EF), were analysed as described previously [6].

In patients, LGE was obtained 12–15 min after intravenous injection of 0.2 mmol/kg Gadobutrol (Gadovist®, Bayer HealthCare, Leverkusen, Germany). An inversion recovery two-dimensional SSFP sequence (FOV adjusted individually; matrix size, 204 × 256; TE, 1.2 ms; TR, 3.0 ms; time of inversion [TI] determined individually; flip angle, 40°) was used nulling the signal of normal myocardium [26]. The evaluation of LGE (presence or absence) as a marker for myocardial fibrosis was performed by two independent radiologists with extended experience in the field.

### Cardiac MRS

**Fig. 1:**
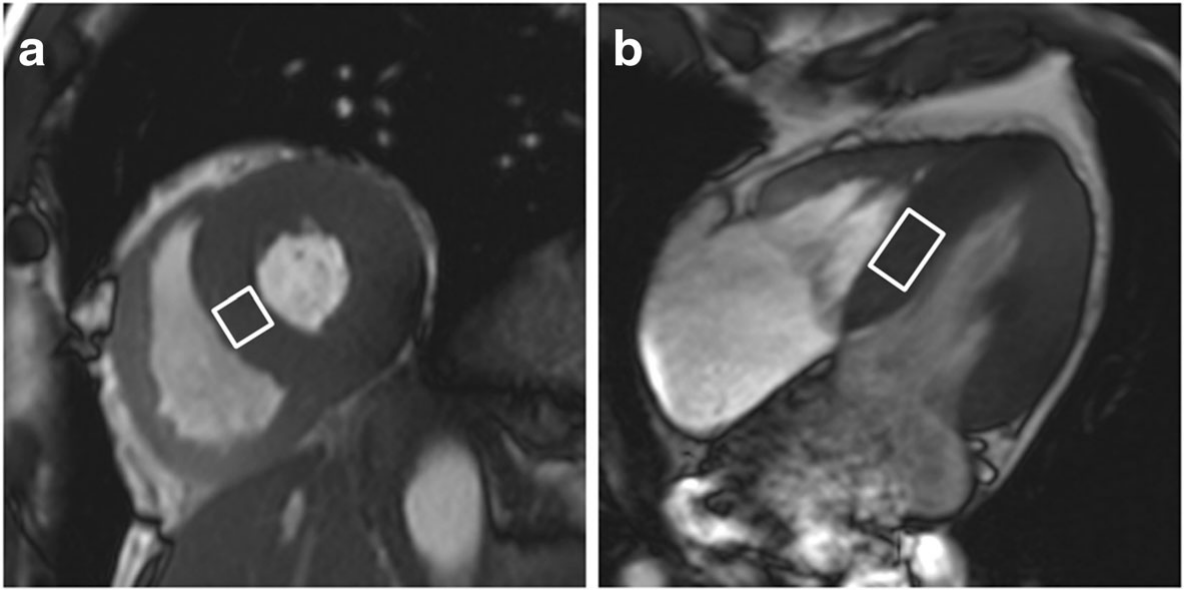
Illustration demonstrating the voxel position (white rectangle) in the interventricular septum on (**a**) short heart axis view and (**b**) four-chamber view at end-systole

**Fig. 2:**
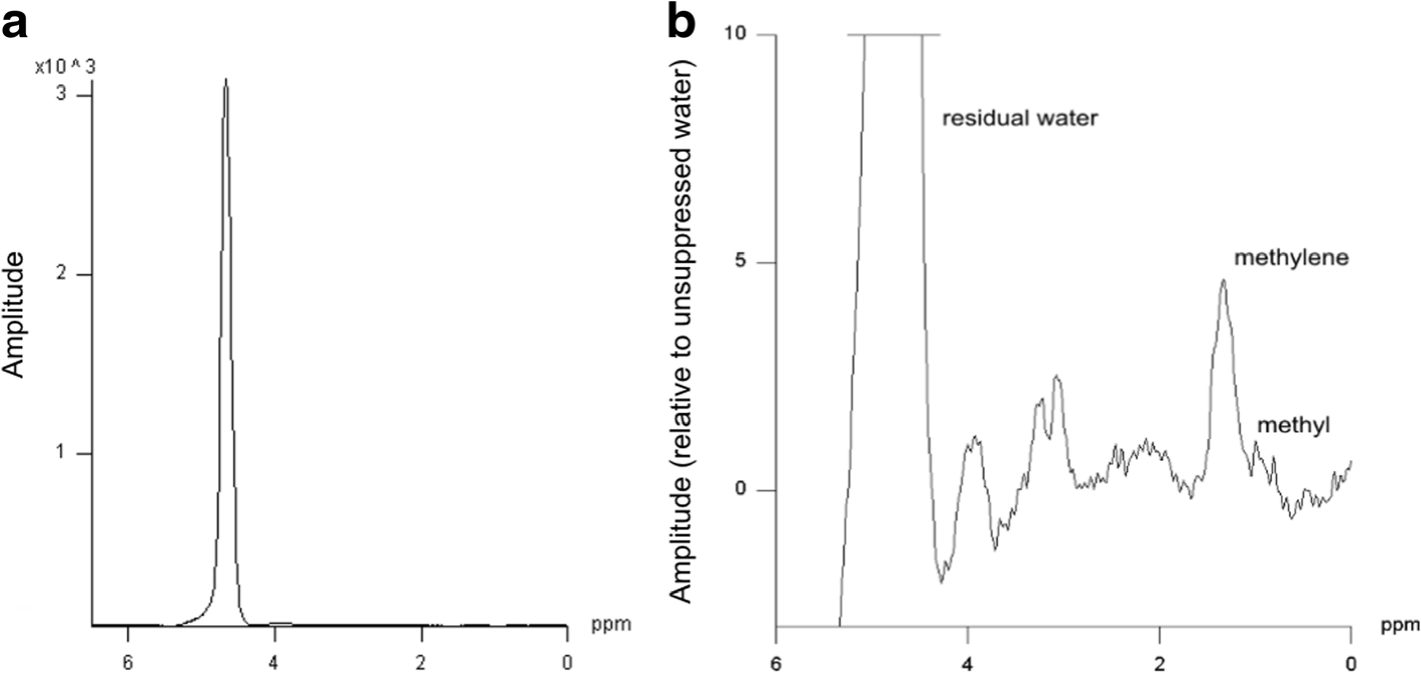
Non-suppressed spectra (**a**) show the typical peak of tissue water set to 4.7 ppm. Water supressed (**b**) single voxel spectroscopy demonstrating lipid peaks in typical position (CH_2_ [methylene] group at 1.3 ppm; CH_3_ [methyl] group at 0.9 ppm). Note the different scaling of the y-and x axis

For data acquisition, a double-triggered single voxel spin-echo spectroscopic sequence (PRESS) was applied (TR of at least one heartbeat [typically one respiratory cycle]; TE = 35 ms; 32 averages). The utilized sequence is a WIP sequence of Siemens® (Siemens AG Healthcare Sector) and not commercially available. Proton spectra were collected from a 20 × 15 × 20 mm^3^ voxel which was positioned within the interventricular septum on the four-chamber and short-axis images, thereby avoiding contamination by epicardial fat (Fig. 1a and b) [27]. For respiratory motion gating a navigator was positioned on the lung-liver interface of the right diaphragm [25]. Measurements were acquired at end-systole and end-expiration. In all subjects, two consecutive ^1^H MR spectra (with and without water suppression) were acquired. As the water signal (set as reference to 4.7 ppm) is the most prominent peak in non-water suppressed spectra (Fig. 2a), the quantification of resonance signals arising from CH_2_ (methylene groups) and CH_3_ (methyl groups) is limited by the digital resolution. The resonance signals of interest become the most prominent peaks in water suppressed spectra. Thus, quantification is more reliable in these data sets. The acquired ^1^H MR spectra with suppression of the water signal revealed the expected resonances for intramyocardial lipids at 1.3 ppm for methylene groups, respectively 0.9 ppm for methyl groups (Fig. 2b).

A vendor specific software (Siemens Sector Healthcare, Erlangen, Germany) was used for post-processing of ^1^H-MRS data. In both types of spectra, post-processing was applied with a Hanning apodization of 300 ms and automatic frequency shift correction. Determination of integral values for the resonance signals was supported by prior knowledge. The water signal was set to a ∂-value of 4.7 ppm, which caused protons in methylene (CH_2_) groups to appear at 1.3 ppm, as well as protons in methyl (CH_3_) groups at 0.9 ppm [28]. The total myocardial lipid content resulted from the addition of the area of the resonance signal of both peaks and was expressed as lipid/water resonance ratio (%).

### Statistical data analysis

Normal distribution of our data was evaluated by the Kolmogorov-Smirnov test and the Shapiro-Wilk test. As EF and LV mass proved to be normally distributed, an independent *T*-test was used for evaluation, while the Mann-Whitney-*U*-Test was utilized for evaluation of the non-normally distributed myocardial lipid content to check for statistical significance. A value of *p* < 0.05 was considered significant. Statistical analyses were performed by using statistical software (IBM SPSS Statistics for Mac, Version 21.0, Armonk, NY, USA).

## Results

### Patients cohort – control group

Gender and age-specific attributes, as well as clinical and laboratory characteristics of the patients cohort are given in Table 1. In particular, the duration of ERT, creatinine blood concentrations and presence or absence of LGE are documented at the time of investigation. Since Fabry disease is known to affect the kidneys, among others, serum creatinine values and glomerular filtration rates of all patients are listed at the time of examination. 16 patients complained of neurological symptoms, such as acroparesthesia. Furthermore, one of these patients (manifest Fabry cardiomyopathy, LGE positive) with prolonged history of the disease suffered a stroke at young age. Mostly, blood pressure was within the norm (range 120/60–155/95 mmHg). However, one overweight LGE positive female patient (BMI 37.7) presented with elevated blood pressure of 178/88 mmHg.

**Table 1:** Clinical and laboratory characteristics of the patients group (*n* = 30)

Patient	age [years]	gender	BMI [kg/m2]	SBP [mmHg]	DBP [mmHg]	ERT [years]	GFR [ml/min]	Crea. [mg/dl]	EF [%]	LVM [g]	LGE	lipids [%]	NS
1	49	f	33.8	140	100	0	113	0.6	55	92	y	0.27	n
2	43	f	20.6	125	60	0	116	0.6	64	127	y	3.29	y
3	17	m	20.0	138	73	1	156	0.7	56	131	n	0.15	y
4	52	f	23.5	170	70	7	82	0.8	70	197	y	5.14	y
5	23	m	29.2	120	78	4	127	0.8	64	217	n	1.76	n
6	43	m	28.7	125	83	9	47	1.7	71	207	y	0.10	y
7	50	f	23.3	104	69	3	70	0.9	78	161	y	0.14	n
8	37	f	27.3	115	85	0	120	0.6	59	104	n	0.30	n
9	51	f	21.6	101	74	3.5	80	0.8	69	78	y	1.36	n
10	19	m	20.2	108	88	3.5	116	0.9	53	129	n	0.61	n
11	27	f	20.5	115	80	0	91	0.8	65	88	n	4.05	n
12	50	f	37.7	178	88	9	94	0.7	79	189	y	0.92	y
13	52	f	38.7	145	95	9	93	0.7	83	175	y	0.85	y
14	43	m	19.2	120	70	10	47	1.7	60	227	y	0.54	y
15	53	f	22.8	120	80	4	76	0.8	73	132	y	0.22	n
16	50	m	17.5	110	80	7	95	0.9	69	253	y	0.30	y
17	42	m	26.9	140	85	7	95	0.9	47	386	y	0.24	y
18	48	f	23.1	110	75	0	86	0.8	69	192	n	0.23	n
19	17	f	19.4	108	76	0	---	0.8	56	83	y	3.59	n
20	68	f	19.8	115	80	0	82	0.7	66	84	n	0.90	y
21	46	m	25.4	129	70	9	69	1.2	65	226	y	0.14	y
22	28	m	31.0	115	65	6	158	0.6	57	155	y	0.10	y
23	50	f	27.2	112	104	0	71	0.9	68	141	y	0.57	n
24	30	m	24.3	135	90	4	112	0.9	69	176	y	0.45	y
25	42	m	21.0	155	95	6	96	0.9	52	179	y	0.10	y
26	22	f	19.3	116	67	0	111	0.7	69	73	n	0.10	n
27	61	f	18.9	140	66	0	77	0.8	61	87	n	0.70	y
28	51	f	21.4	91	69	0	70	0.9	69	140	y	0.18	n
29	51	f	28.4	145	95	0	69	0.9	56	148	y	0.16	y
30	28	m	24.2	133	70	9	90	1.0	55	203	n	0.36	n

*BMI* body mass index, *SBP* systolic blood pressure, *DBP* diastolic blood pressure, *ERT* treatment with enzyme replacement therapy, *GFR* glomerular filtration rate, *Crea* serum creatinine concentration, *EF* ejection fraction, *LVM* left ventricular mass, *LGE* late enhancement, *NS* neurological symptoms

#### Spectroscopic measurements

**Fig. 3:**
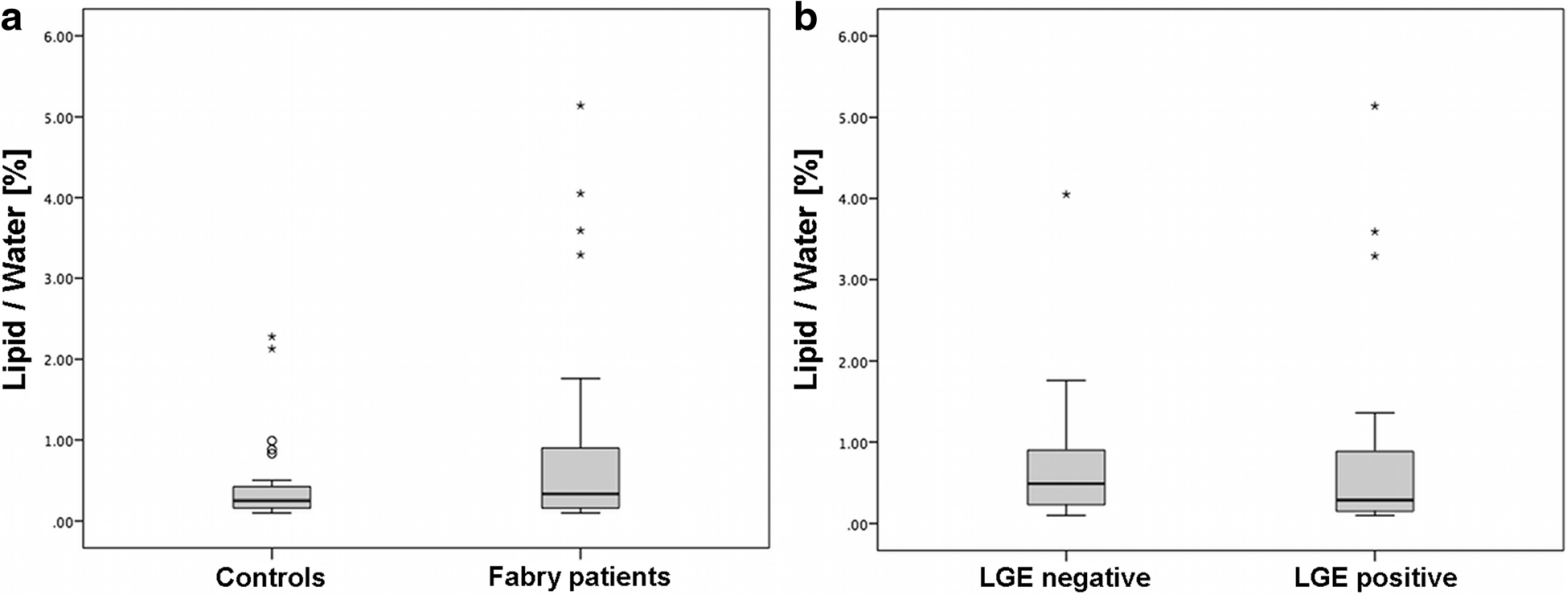
Boxplots demonstrate that myocardial lipid content was not significantly elevated in Fabry disease (*p* = 0.225). Nevertheless four Fabry patients presented with a notable myocardial lipid content of > 3.0 % (**a**). Within the patient cohort myocardial lipid content is similar in late gadolinium enhancement (LGE) positive and LGE negative Fabry patients (*p* = 0.509) (**b**)

Myocardial lipid content was not significantly elevated in Fabry disease (*p* = 0.225), although the lipid/water resonance ratio tended to be higher in Fabry patients (median 0.33 %; IQR 0.16–0.91 % [range 0.10–5.14 %]; mean ± standard error (SE) 0.93 ± 0.24 %), as compared to the healthy control group (median 0.25 %; IQR 0.16–0.42 % [range 0.10–2.28 %]; mean ± SE 0.44 ± 0.10 %) (Fig. 3a). Four female Fabry patients presented with a notable myocardial lipid content of > 3.0 % (3.29, 5.14, 4.05, 3.59 %; see Table 1 for detailed patients information) whereas only two controls presented with myocardial lipid content of slightly above 2.0 % (2.13 %, 2.28 %).

#### Parameters of cardiac function

Measurement of LV mass differed significantly in both groups. Compared to the healthy control group (mean ± SD; 127.6 ± 27.1 g), LV mass was significantly higher in Fabry patients (mean ± SD; 159.3 ± 66.5 g); (*p* = 0.019) (Fig. 5a). In contrast to LV mass parameters, EF was similar in both groups (mean EF ± SD; 64.2 ± 8.6 % in patients vs. 63.2 ± 6.5 % in controls) (*p* = 0.613).

### Patients LGE positive – Patients LGE negative

**Fig. 4:**
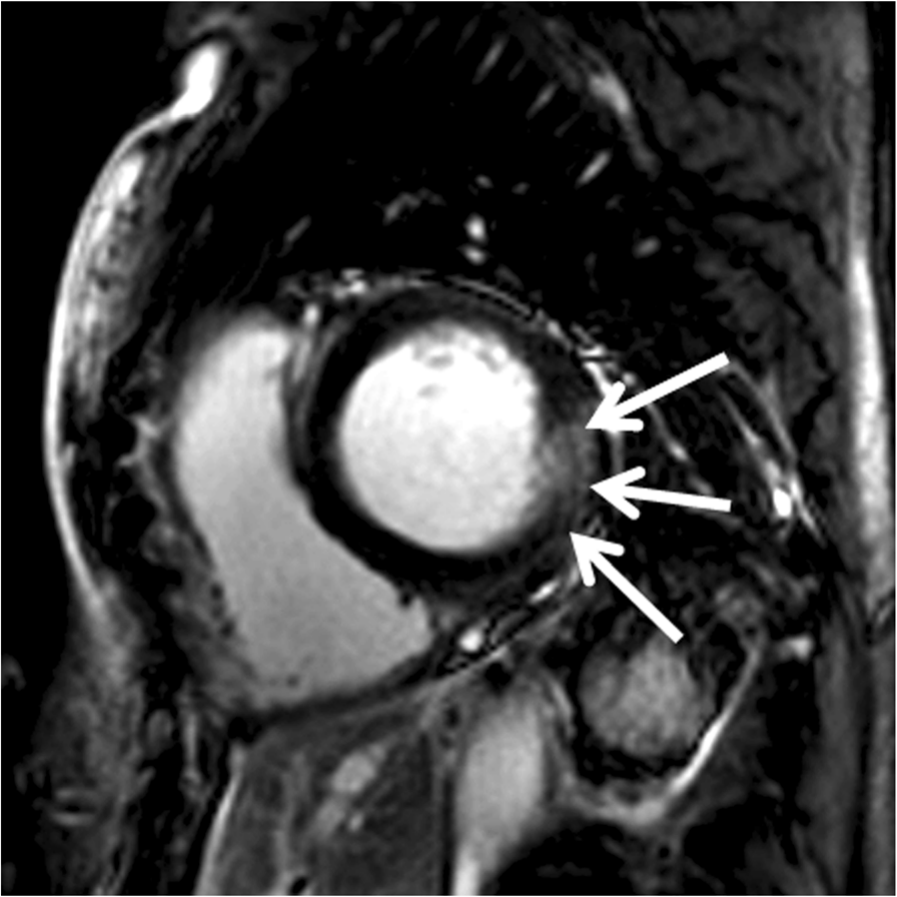
Late gadolinium enhancement (LGE) in the typical position for Fabry disease in the inferolateral parts of the left ventricle (*arrows*) of a 43 year-old male patient

In 20 out of 30 patients LGE was found in the basal inferolateral section of the left ventricular wall (Fig. 4).

#### Spectroscopic measurements

Within the patients cohort the comparison of mean lipid/water ratio in LGE negative and LGE positive Fabry patients revealed similar myocardial lipid content in both subgroups (median 0.29 %; IQR 0.15 – 0.90 % [range 0.10–5.14 %]; mean ± SE 0.93 ± 0.32 % in LGE_pos._ vs. median 0.49 %; IQR 0.21–1.12 % [range 0.10–4.05 %]; mean ± SE 0.92 ± 0.36 % in LGE_neg._) (Fig. 3b), showing no significant difference (*p* = 0.509).

### Parameters of cardiac function

**Fig. 5:**
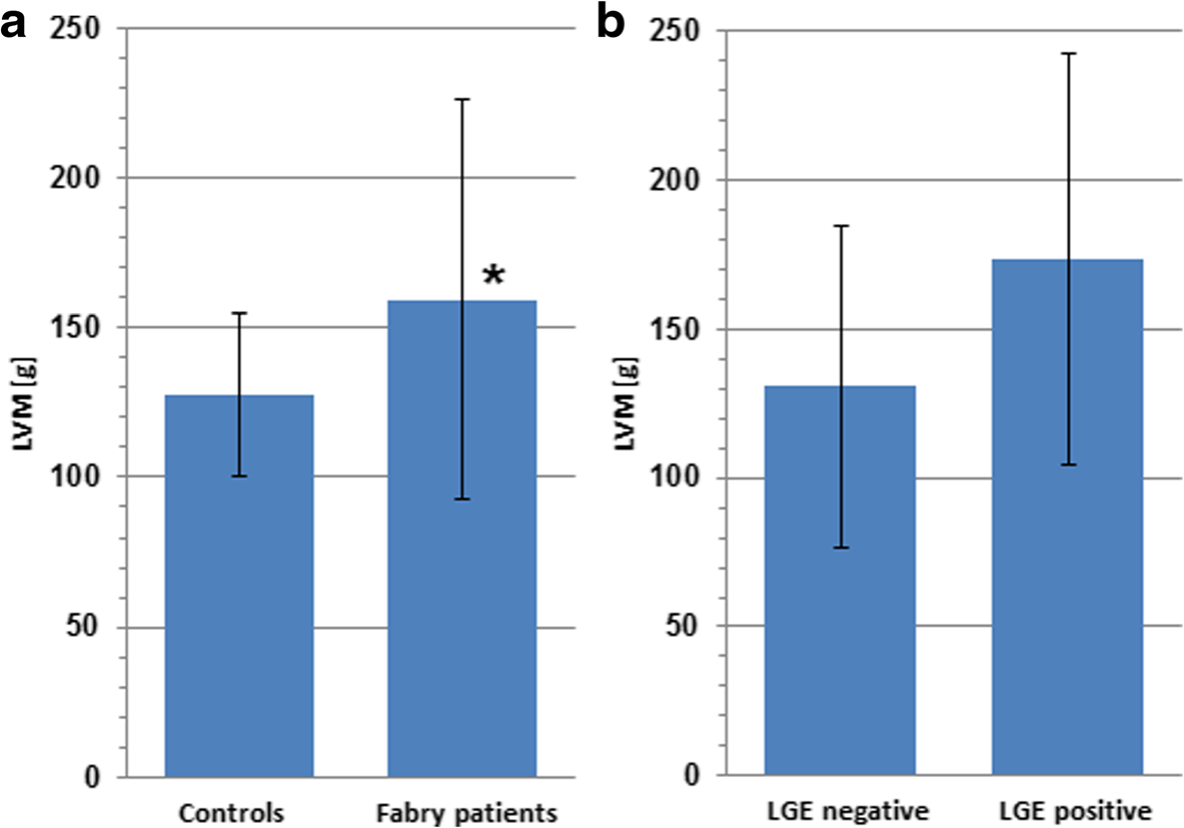
Left ventricular mass (LVM) is significantly elevated in Fabry disease (* *p* = 0.019) (**a**). Within the patient cohort LVM is tendentiously higher in late gadolinium enhancement (LGE) positive Fabry patients (*p* = 0.076) compared to LGE negative patients (**b**). Error bars show standard deviation

Compared to LGE negative (*n* = 10) patients, we observed a trend towards LV mass increase in LGE positive (*n* = 20) Fabry patients (mean LV mass of 173.6 ± 68.9 g in LGE_pos._ vs. 130.8 ± 54.2 g in LGE_neg._ patients) (Fig. 5b) without statistical significance (*p* = 0.076). LV function (expressed as EF) was similar in LGE positive and LGE negative Fabry patients (mean EF ± SD; 65.5 ± 9.5 % in LGE_pos._ vs. 61.7 ± 5.8 % in LGE_neg._) (*p* = 0.188).

## Discussion

### Cardiac involvement in Fabry disease

With progress of the disease, almost all male Fabry patients develop symptoms of cardiac insufficiency due to pathological alteration of cardiac morphology and function [29]. Increase of myocardial mass and progressive myocardial fibrosis are the hallmarks of cardiac involvement in Fabry disease and can be detected in MR imaging [7, 8, 30]. While the deposition of Globotriaosylceramide (Gb3) is ubiquitous in the myocardium, LGE is typically restricted to the inferior basal parts of the left ventricle [7].

Accordingly, 20 of our 30 patients presented LGE in the inferolateral parts of the left ventricular wall, corresponding with the typical localization of fibrosis in Fabry cardiomyopathy. One might argue that myocardial lipid acquisition and, respectively, voxel placement should be chosen in a position typical for LGE, improving the likelihood of acquiring even higher pathological lipid values. Taking into account that patients suffering from Fabry disease develop a global left ventricular hypertrophy in late stages of the disease and ubiquitous deposition of sphingolipids in the myocardium of patients suffering from cardiac Fabry disease is described in literature, we assume a change of lipid concentration spread over the whole myocardium. There is an appealing advantage of voxel placement in the interventricular septum: there is only a minor or even zero risk to acquire false positive results deriving from lipid contamination of the voxel by epicardial fat [25, 27, 31–36].

Because of the coexistence of advanced renal disease in Fabry patients, which sometimes precludes the use of a contrast agent, retention parameters (glomerular filtration rate, serum creatinine) were collected from all patients. Furthermore most of the patients had normal blood pressure values. Only one female presented with an elevated blood pressure of 178/88 mmHg. Possible explanations might be the patient suffering from Fabry cardiomyopathy (LGE positive; elevated LV mass), or her advanced obesity (BMI 37.7).

### Cardiac ^1^H-MRS

Although we are aware that methylene (CH_2_) and methyl (CH_3_) groups do not represent the total myocardial lipid content, we limited our measurements to these two peaks in accordance with previous studies [17, 25]. The mean lipid/water resonance ratio of 0.44 ± 0.09 % detected in healthy subjects is in accordance with previously published data ranging from 0.38 ± 0.02–0.52 ± 0.11 % [17, 25, 37].

Our results show no significant difference between myocardial lipid content in Fabry patients and healthy controls (*p* = 0.225). For the majority of Fabry patients there was no evidence that lysosomal storage of sphingolipids influences cardiac lipid content as measured by ^1^H-MRS. However, we found noticeably elevated myocardial lipid content in a small number of female Fabry patients (4 patients [3 LGE positive] with a lipid/water resonance ratio > 3 %), which raises the question of potential gender effects. The average cardiac lipid content found in our Fabry cohort is not in line with former study results by Thompson et al. postulating elevated cardiac lipid content in a relatively small cohort of *n* = 4 Fabry patients [13]. Furthermore, a previous study of Fabry disease from our own research group measures myocardial lipid elevation only in one patient presenting with advanced Fabry cardiomyopathy, whereas the average number of Fabry patients in this study presented with normal lipid-to-water-ratio [17].

Conceivable explanations for higher cardiac lipid content measured in a few subjects from the Fabry cohort may be firstly an applied ERT for an extended time period (mostly between 4 and 10 years; select patients between 1 and 3.5 years), indicating a prolonged disease history. On the other hand, the potential of ERT to affect myocardial lipid content has not been addressed in literature. Secondly, the published cohort gender proportion is imbalanced towards females (12 male, 18 female). Consequently, a necessary consideration includes the lack of correlation of loss of myocardial function and fibrosis with myocardial hypertrophy among females as opposed to males [4]. Furthermore, gender specific relative elevation of myocardial lipid content in females has been elucidated in the past [38], an assumption which could possibly influence our findings.

The subgroup evaluation of LGE positive and LGE negative patients within the Fabry cohort revealed a trend towards elevated LV mass in LGE positive patients (*p* = 0.076). When evaluating LGE positive and negative subgroups within the Fabry cohort, no correlation between the presence of LGE (typically occurring in the lateral wall) and septal lipid concentration measured by ^1^H-MRS was observed. Our results suggest that there are at least two different underlying pathomechanisms in Fabry cardiomyopathy. Time line development of fibrosis and elevated myocardial lipid content occurring in some individuals seems unpredictable at this time, possibly due to differences in underlying pathomechanisms.

Most studies concerning ERT in Fabry disease demonstrate a decelerated development of disease after early ERT onset. Therefore early detection of myocardial changes is from utmost importance. Including the results of Sado et al., who suggested myocardial T_1_ values as a marker of early cardiac involvement [14], a comprehensive cardiac examination including three subsections (LGE; ^1^H-MRS; T_1_ mapping) could hold the highest potential for the final assessment of early and late myocardial changes, including the potential role of lipid accumulation in cardiac Fabry disease.

### Limitations

Due to the known risks of myocardial biopsy, we did not perform comparative invasive triglyceride measurements. Previously published data by O´Connor et al. demonstrated a remarkably high correlation coefficient between in vivo (^1^H-MRS) and ex vivo triglyceride measurements [21]. The results of that study suggest that triglyceride quantification by MRS is suitable for clinical routine studies.

At the time of examination 18 patients were treated with ERT, while 12 patients did not receive ERT. The influence of ERT on myocardial lipid content remains unclear to date. A separate statistical evaluation of patient subgroups with and without ERT was not performed within this study cohort due to sample size. Nevertheless, long-term monitoring of myocardial lipids using ^1^H-MRS to explore the effects of ERT represents an interesting field for larger future studies.

We did not apply intravenous contrast medium to our control group, while this was used in the patient cohort. To ensure a short examination time and to avoid an artificial prolongation of the investigation, the time slot between i.v. contrast application and LGE acquisition was used for the spectroscopic measurements in our patients. One must be aware that gadolinium-based contrast agents potentially increase the water peak area, as well as selected metabolite peaks (e.g. choline), especially when using short echo times [39]. Particularly these observations are described in breast cancer as well as brain tumors [40–43]. However, best to our knowledge, there is no published data showing an influence of gadolinium based contrast agents on the results of lipid evaluation in cardiac ^1^H-MRS. In the future, upcoming single breath hold ^1^H-MRS sequences should enable time-saving spectroscopic data acquisition in patients prior to i.v. contrast administration [44].

Furthermore, mean age comparison between patients and controls is not fully congruent. More importantly, in the light of the knowledge that BMI correlates with myocardial lipid content [37], the mean BMI of the two cohorts shows a slight mismatch (mean BMI in patients 24.5 kg/m^2^ vs. 22.4 kg/m^2^ in controls; *p* = 0.079). We cannot exclude any influence of the BMI on lipid levels within our patients. Moreover, the gender proportion of the patient cohort is imbalanced towards females (12 male vs. 18 female).

Owing to the x-linked inheritance of Fabry disease, a separate evaluation of both genders will be necessary in future studies. Due to the sample size this was not feasible in the present study. Therefore, further larger studies will be needed to elucidate possible gender aspects.

## Conclusions

The present study demonstrates the potential of lipid metabolic investigation embedded in a comprehensive examination of cardiac morphology and function in Fabry disease. There was no evidence that lysosomal storage of sphingolipids influences cardiac lipid content as measured by ^1^H-MRS. Therefore the potential of ^1^H-MRS as a tool for early diagnosis of cardiac involvement and for optimization of ERT onset as leading therapy in Fabry disease remains questionable and needs to be further evaluated. In future native T_1_ mapping might be a superior comparator to validate actual changes in the myocardium. Finally, the authors share the opinion that a comprehensive cardiac examination including three subsections (LGE; ^1^H-MRS; T_1_ mapping), could hold the highest potential for the final assessment of early and late myocardial changes in Fabry disease.

## Abbreviations

BMI: Body mass index
ECG: Electrocardiogram
EF: Ejection fraction
ERT: Enzyme replacement therapy
FOV: Field of view
IQR: Interquartile range
LGE: Late gadolinium enhancement
LV: Left ventricular
MRI: Magnetic resonance imaging
MRS: Magnetic resonance spectroscopy
SD: Standard deviation
SE: Standard error
SNR: Signal/Noise-ratio
SSFP: Steady-state free precession
TG: Triglycerides
TI: Time of inversion
